# Determination of Egg Number Added to Special Pasta by Means of Cholesterol Contained in Extracted Fat Using GC-FID

**DOI:** 10.3390/foods7090131

**Published:** 2018-08-24

**Authors:** Daniele Naviglio, Ciro Langella, Stefano Faralli, Martina Ciaravolo, Maria Michela Salvatore, Anna Andolfi, Vincenzo Varchetta, Raffaele Romano, Monica Gallo

**Affiliations:** 1 Department of Chemical Sciences, University of Naples Federico II, via Cintia, 4, 80126 Naples, Italy; naviglio@unina.it (D.N.); martinaciaravolo@gmail.com (M.C.); mariamichela.salvatore@unina.it (M.M.S.); andolfi@unina.it (A.A.); vincenzo.varchetta@unina.it (V.V.); 2 Department of Molecular Medicine and Medical Biotechnology, University of Naples Federico II, via Pansini, 5, 80131 Naples, Italy; cirolangella88@gmail.com; 3 Medical Center, Piazzale Luigi Cadorna, 9, 20123 Milan, Italy; info@faralli.net; 4 Department of Agriculture, University of Naples Federico II, Parco Gussone, Portici, 80055 Naples, Italy; raffaele.romano@unina.it

**Keywords:** special pasta, gas chromatography, fats, cholesterol, squalene, lyophilized yolk, egg number

## Abstract

Pasta with eggs added (generally termed “special pasta” for Italian legislation) is made by adding no less than 4 eggs without shells (or no less than 200 g of liquid or lyophilized egg product) per kilogram of semolina, as provided by law. In this work, to determine the final content of eggs added to dough, an analytical procedure was developed for the rapid analysis of the cholesterol content in the finished pastas. The proposed procedure was simpler, faster, and more accurate than that of official methods of analysis based on the gravimetric determination of sterols. Moreover, the determination of the quality of fat content in the special pasta (egg pasta in this case) allowed the evaluation of its origin, avoiding possible fraud resulting from the addition of foreign fat as an alternative to fat derived from eggs. In this new gas chromatographic procedure, the internal standard squalene for the quantification of cholesterol was used because a more polar GC capillary column was used (RTX 65 TG-HT) for the separation of sterols, rather than 5% phenyl methylsilicone. The ratio between cholesterol and squalene allowed for the determination of the number of eggs added, while from analysis of the same gas chromatogram, it was also possible to evaluate the composition of triglycerides in the fat contained in the pasta, allowing discrimination of foreign fats with respect to fats contained in eggs and therefore avoiding adulteration of pasta. The same analytical procedure was applied to the determination of cholesterol content in lyophilized yolk.

## 1. Introduction

Pasta is a popular food throughout the world and its consumption is rapidly increasing worldwide because of the simplicity of its preparation and its pleasant taste. The origins of pasta are very old, and it is unknown where it was first used [1,2,3,4]. According to current legislation, “special pasta” is pasta that, in addition to containing water and durum wheat flour or semolina, makes use of other ingredients (eggs, vegetables, spices, etc.). This must be marketed with the term “pasta made from durum wheat flour or semolina” followed by the ingredient(s) used. In particular, egg pasta is a product obtained from processing dough based on semolina flour and at least 4 whole eggs without shell (or the equivalent in egg liquid product or lyophilized product), with an egg ratio/flour ratio of 1/5 (e.g., 200 g of shell-less egg, or the equivalent in egg liquid product, per 1000 g of semolina). Egg pasta is, therefore, a type of special pasta that meets additional requirements and must be marketed with the term “egg pasta” [5].

The terms egg pasta and fresh pasta are not necessarily synonymous. In practice, egg pasta can be both fresh and/or dried, taking into account that fresh egg pasta can be made at home, in the lab or even on an industrial scale and immediately frozen or immediately used. However, dry egg pasta is sold packaged, similar to semolina pasta. Eggs can be replaced by a corresponding amount of liquid or solid egg product made exclusively from chicken eggs, according to legal requirements [6,7]. Furthermore, the presence of common wheat flours is tolerated at no more than 3% (*w*/*w*). The actual official reference methods for the determination of the number of eggs in egg pastas are essentially based on a determination of the total sterols and ethereal extract fat content [5]. Since the commercial value of egg pasta is related to the number of added eggs, it is very important to have an accurate method for accurately determining egg content.

The determination of cholesterol content allows the determination of the number of eggs added to the pasta because it is present only in eggs (animal fat) and is not present in semolina (vegetable fat). The current legislation does not indicate any instrumental reference method that can be used to ascertain the number of eggs in special pasta to highlight potential fraud. One method, although not official, has been proposed by Muntoni et al. (1966) and is based on determining the relationship between cholesterol and β-sitosterol and/or between cholesterol and campesterol [8] and is used as a reference method. The sterols β-sitosterol and campesterol are naturally present in the bran, while cholesterol is introduced by the addition of eggs to flour. Due to high variability of these vegetable sterols, the method is highly inaccurate. 

Another source of error is that the value of cholesterol content in eggs fluctuates over a relatively wide range, and the average value found in the recent literature was found to be much lower than that determined in the early 1960s [9,10]. In fact, significant experimental data have shown that the total free cholesterol content was in the range 120–193 mg/egg (average value 157 ± 3 mg/egg) [11], lower than the previously reported value of 213 mg/egg [12]. For many years, great attention has been devoted to the identification of the main risk factors for cardiovascular disease, and in this context, several epidemiological studies have demonstrated the existence of a relationship between mortality from cardiovascular disease and cholesterol blood levels [13]. To date, no research has clearly established a link between egg consumption and risk for coronary heart disease and this should suggest the need of a more in-depth reevaluation and reconsideration of the association between the intake of cholesterol-related foodstuff and human health. In addition, data from recent studies show that the consumption of one or two eggs per day, when part of a low-fat diet, does not adversely affect the lipid profile; the other way around, the preclusion of eggs from the diet may represent a potential reduction in the overall dietary quality. However, some recent studies have highlighted that there is no risk to human health, even assuming a consumption of 2–3 eggs per day, which was contrary to what had been accepted for many years [14,15,16,17]. In truth, the consumption of up to 3 eggs per day resulted in an overall beneficial effect in terms of the biomarkers associated with CVD risk [18]. The determination of cholesterol content in egg pastas is important only for commercial aspects, and not for human consumption because it is not a concern for human nutrition (USDA guidelines). In addition, eggs can be an important food source for improving the plasma carotenoid status in a population at high risk for cardiovascular disease and type 2 diabetes [19]. 

Moreover, since a freeze-dried egg product is used in the production of egg pasta, it is necessary to know the number of eggs used in the freeze-dried product and to determine the correct cholesterol content, starting from a fixed product, to determine the correct addition of the lyophilized product. However, it was not easy to obtain this information on an industrial level; thus, it was necessary to establish an average value of cholesterol per egg that was close to the results of the analysis conducted using predetermined egg pasta and the desired number of eggs that the producer intended to add to the semolina.

The first aim of this study was to provide operators in the egg pasta industry with a standardized procedure, based on gas chromatography equipped with FID (flame ionization detector), to enable determination of the final content of eggs added to the dough via rapid analysis of cholesterol content, either in the finished pastas or in the egg product. In addition, the proposed procedure could be useful for the control of authenticity of added fat to assure the addition of eggs alone to semolina, avoiding possible adulterations.

Finally, and more generally, given its importance from a health perspective, another aim was to obtain a rapid procedure for the determination of cholesterol in different food matrices.

## 2. Materials and Methods

### 2.1. Equipment

A Waring blender (New Hartford, CT, USA) with a 0.106-mm sieve, vacuum chamber, a 28 cm-long glass column with a porous separator and 3.5 cm diameter, and a nitrogen cylinder were used. The standards, reagents and solvents used were all of analytical grade. The standards cholesterol, squalene beta-sitosterol, cholestanol, n-octacosane, mono-, di-, and triglycerides kit; diethyl ether, anhydrous sodium sulphate were purchased from Sigma-Aldrich (Milan, Italy).

Instrumentation: An Autosystem XL gas chromatograph (Perkin Elmer, Norwalk, CT, USA) equipped with a programmed split-splitless (PSS) injector and flame ionization detector (FID) and connected to a Turbochrom version 4.1 data acquisition system was used for cholesterol analysis. For the separation steps, a PK 131 centrifuge (ALC International, Milan, Italy) and a rotary evaporator (Heidolph, Laborota 4000, Schwabach, Germany) were used.

### 2.2. Sampling

Durum wheat pasta samples used for experimentation were four different Italian brands and were purchased at local markets. The production date and percentage of egg content added for each pasta sample were recorded. First, 250 g of each commercial egg paste sample was homogenized in a Waring Blender at 24,000 rpm. Subsequently, the powered samples were dried in an oven at 50 °C for 12 h before being subjected to the cholesterol extraction procedure. Two lyophilized yolk samples (A and B) purchased at the market were analysed following the same procedure. Four standard samples of semolina were added with a measured quantity of cholesterol and vegetable olive oil to prepare four standard samples of pasta. Standard samples of semolina received 300, 600, 900 or 1200 mg cholesterol per kg and were analysed for recovery. These samples were analysed in triplicate to determine the recovery of cholesterol.

### 2.3. Preparation of Samples for Analysis

#### 2.3.1. Analysis of Pasta Samples

**Figure 1:**
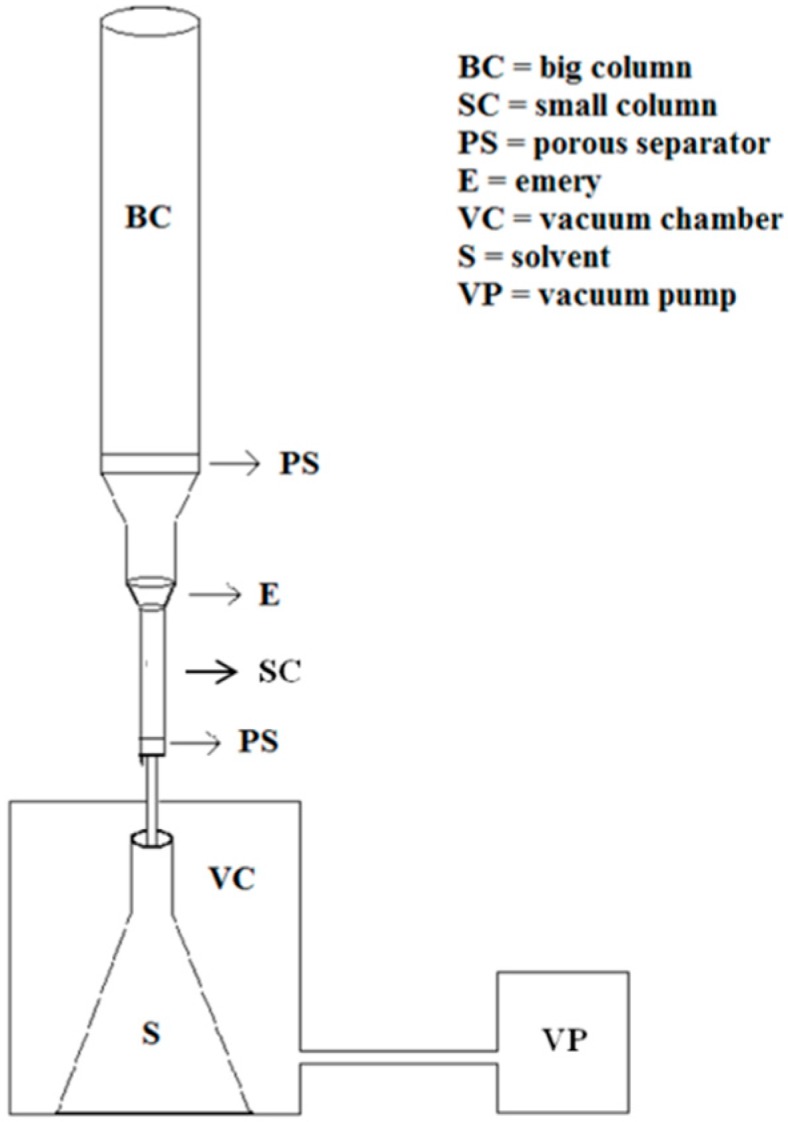
System for rapid extraction of fat from powered pasta and lyophilized egg.

Pasta (100 g) was ground in a Waring blender and sifted through a 0.106-mm sieve. A column with a filter at the base was filled with the sieved pasta to approximately one-quarter of the total volume and attached to the vacuum chamber; a container was inserted under the column in the vacuum chamber, and the system was closed to activate the vacuum. Percolation of solvent through the column is an effective and rapid extraction technique that produces rapid, high-yield recovery and allows the sample concentration required for an accurate quantitative analysis. A glass column with a porous septum was attached to the end of the major column containing anhydrous sodium sulphate. At this point, the system was ready; the vacuum was activated, and 100 mL of ethyl ether was added to the column containing pasta (Figure 1). The fat was eluted with 100 mL of diethyl ether, which was then passed through sodium sulphate to remove water residue. The resulting solution was evaporated to dryness on a rotary evaporator, and the last traces of solvent were removed with nitrogen gas. An aliquot of 50 mg of fat was weighed on an analytical balance, and exactly 1 mL of squalene solution (3000 ppm) was added as an internal standard (IS). The solution was vortexed until complete dissolution of the fat, and 0.5 μL of the solution was injected into the gas chromatograph. Finally, the areas of the peaks of squalene, cholesterol and β-sitosterol were integrated in the gas chromatogram. The standard samples of pasta were analysed following the same procedure reported for the pasta.

#### 2.3.2. Analysis of Lyophilized Yolk

For the analysis of cholesterol content in lyophilized yolk, 10 g of powder was passed through a column under vacuum that was approximately 25% filled relative to the total volume. The fat was eluted with 100 mL of diethyl ether that was previously passed through sodium sulphate to remove water residue. The resulting solution was evaporated to dryness on a rotary evaporator, and the last traces of solvent were removed with nitrogen gas. An aliquot of 50 mg of fat was weighed on an analytical balance, and exactly 1 mL of squalene solution (3000 ppm) was added as an internal standard (IS). The solution was vortexed until complete dissolution of the fat, and 0.5 µL of the solution was injected into the gas chromatograph. Finally, the areas of the peaks for squalene, cholesterol and β-sitosterol were integrated in the gas chromatogram. The extracts were quantified with known quantities of standard compounds.

### 2.4. Chromatographic Conditions

GC conditions: Column: 65% diphenyl 35% dimethyl polysiloxane stationary phase, RTX 65 TG-HT (Restek, Bellefonte, PA, USA); L = 30 m; i.d. = 0.25 mm; f.t = 0.10 μm. Injector program: 70 °C for 12 s, increase at 999 °C/min to 370 °C, hold for 5 min. Column program: 220 °C for 2 min, increase at 5 °C/min to 360 °C, hold for 5 min. FID temperature: 370 °C. Carrier gas: hydrogen; flow rate: 1.5 mL/min and 2.0 mL/min; Split ratio: 1:80.

The reset system of the drift (background) due to the increased temperature was used and calibrated with three acquisitions of the baseline signal to enable better integration of the triglycerides [20].

### 2.5. Analytical Validation

The calibration curve was prepared by adding, to a solution of cholesterol, a solution of squalene in ethyl ether (3 mg/mL or 3000 ppm) as an internal standard (IS). A calibration curve for cholesterol was prepared by weighing aliquots of 10, 15, 30, 45 and 60 mg of cholesterol on an analytical balance and transferring them to three 10-mL volumetric flasks. Then, the ethyl ether solution of squalene (3 mg/mL) was added to the mark. The concentrations of the prepared solutions were 1000, 1500, 3000, 4500 and 6000 mg/L for cholesterol, while that of the IS was always 3000 ppm. The linear range was between 0 and 2000 ppm; the limit of detection was 10 ppm, and the precision was 5%. An aliquot of 0.5 μL of each solution was injected into the gas chromatograph, and the gas chromatograms were acquired. The ratios between the areas of the peaks of cholesterol and squalene (IS) obtained for the three solutions according to their cholesterol concentrations were recorded in a graph. The experimental points obtained under these analytical conditions were modelled by the function *R* = 0.000242 × *C*, where R represents the value of the ratio between the area of the peaks of cholesterol and the area of internal standard, whereas *C* represents the concentration of cholesterol (ppm) [11].

## 3. Results and Discussion

### 3.1. Determination of the Number of Eggs According to Muntoni’s Method

The reference methods for the determination of the number of eggs in egg pasta were essentially based on measurement of the total sterol content because cholesterol is an animal steroid found in eggs, while β-sitosterol and campesterol are vegetable steroids present in flour and semolina. The current legislation does not indicate any instrumental reference method for determining the number of eggs in special pasta or any analytical procedure to identify potential fraud. The Muntoni method, although it is not an official method, is taken as reference method for the determination of egg number in pasta and is based on the determination of the ratio between cholesterol and β-sitosterol and/or between cholesterol and campesterol [8]. This method involves saponification of the sample, which is necessary to transform the triglyceride component of the lipids in the sample, which is not easily detectable on a non-polar column, into fatty acids. In the past, a review by Christie (1993) reported the principles behind the more important esterification and transesterification procedures, their advantages and disadvantages and their applications to various classes of lipids [21]. The method proposed in this paper was also used to tentatively determine the number of eggs added to the pasta according to Muntoni et al. (1966) [8]. In another work, it was demonstrated that it was possible to apply such a ratio even in the analysis of fat components on a capillary column [22]. Furthermore, since it has been demonstrated that the cholesterol content in eggs was not increased by the transesterification reaction [23,24], as compounds such as cholesterol esters are not present in appreciable amounts, the determination of the free cholesterol content in the egg pasta remains rigorous. However, the presence of esters of β-sitosterol and campesterol cannot be considered negligible; in fact, they comprise approximately 5% by weight of the fat extracted from semolina and can contribute to free sterols following the transesterification of fat. For this reason, this process increases the variability of the content of sterols and thus the errors in the assessment of the relative ratios between cholesterol and the other sterols.

Analysis was conducted using two procedures (data not shown), both of which showed a percentage deviation that exceeded 30% and deviations from the true value that were as high as 40%. These results indicated that the number of eggs was underestimated by an amount equal to almost half of the number added, which was due to the high average value of cholesterol content in the eggs used as a reference. To date, in the literature, some work has already been reported on the determination of egg content in egg pasta samples [25,26,27,28], although each method provides an estimate of this number. The reason for this limitation lies in the fact that there has been great variability in the different parts that constitute the egg and its cholesterol content [11,29]. In this perspective, the method presented in this work has allowed the determination of not only the cholesterol content but also, when necessary, the composition of the fatty component triglycerides contained in the paste in order to avoid fraudulent addition. Knowledge of the content of cholesterol in a consumer food such as pasta is also important to help control the intake of cholesterol. In fact, it is well known that a balanced diet and/or a diet supplemented with active ingredients provides valuable aid in the prevention of cardiovascular diseases and metabolic disorders [30]. There are many reports on cholesterol determination by GC analysis [31,32,33,34], but the proposed method is interesting because it includes direct analysis of the lipid fraction, as all of the cholesterol in the egg is present in a free form. However, the presence of esters of β-sitosterol and campesterol cannot be considered negligible; in fact, they make up approximately 5% by weight of the fat extracted from semolina and can contribute to the free sterols after transesterification of fat. For this reason, this process increases the variability of the sterol content and thus increases the errors in the assessment of the relative ratios between cholesterol and other sterols.

### 3.2. Determination of the Number of Eggs According to the New Method

The official method for the extraction of the fat component from flour requires either batch extraction in ethyl ether for twenty-four hours or Soxhlet extraction. In this study, a rapid system based on ethyl ether percolation through flour packed in a glass column was set up and then compared with the official methods (data not shown). The results obtained revealed that passage through a 0.106-mm sieve allowed recovery of the fraction of the flour that was subjected to the extraction column and extracted 98% of the fat contained in the flour, thus representing a good compromise between the recovery efficiency of the total fat and reduced analysis time. In comparison, passage through a sieve of 0.070 mm provided a recovery of 100% but significantly increased the analysis time (Figure 1).

**Figure 2:**
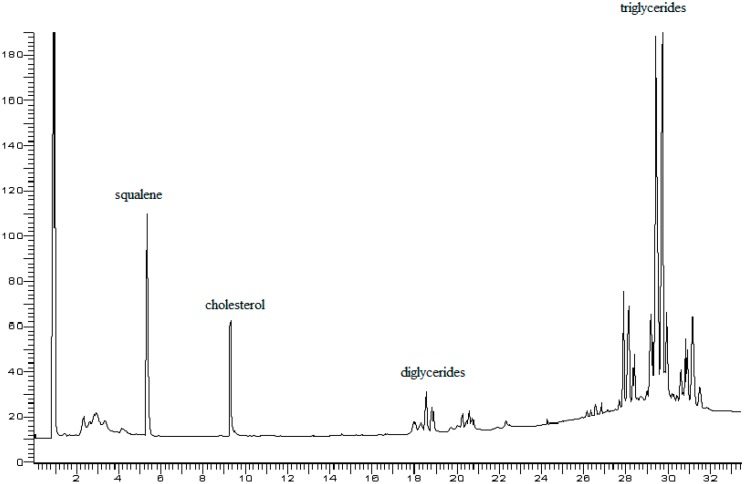
Gas chromatogram of fat extracted from lyophilized egg yolk with the addition of squalene as an internal standard (IS). Carrier flow: 1.5 mL/min.

**Figure 3:**
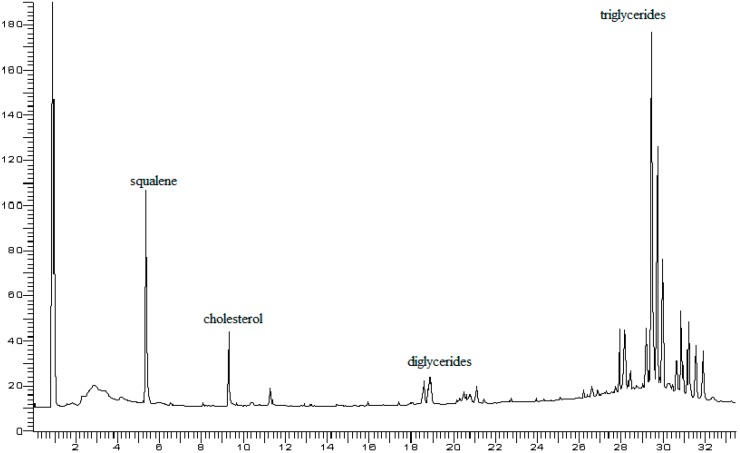
Gas chromatogram of fat extracted from egg pasta with addition of squalene as an internal standard (IS). Carrier flow: 1.5 mL/min.

The gas chromatograms reported in Figure 2 and Figure 3 show very good separation of the internal standard squalene from cholesterol; the triglyceride compounds derived from the fat of eggs are also completely separated. The analysis of the triglyceride component was performed only on a qualitative level and highlighted that only comparison with egg fat was made, but this qualitative comparison was sufficient to identify the native distribution of triglycerides in egg fat. On the other hand, the identification of triglyceride peaks could be achieved by comparison to a recently published work on the triglycerides of butter [35]. In addition, from the analysis of triglycerides, it was possible to obtain more information about the actual addition of egg fat. Moreover, it was possible to compare the profile of the triglyceride components in the fat extracted from egg yolk (Figure 2) to values typical of egg pasta (Figure 3). This type of control would be useful to detect any fraud in the preparation of egg pasta, in which a quantity of foreign fat is added to increase the cholesterol content compared to that derived only from the egg. This determination could not be obtained with the ponderal analysis provided by the reference methods or with application of Muntoni’s method [8].

**Figure 4:**
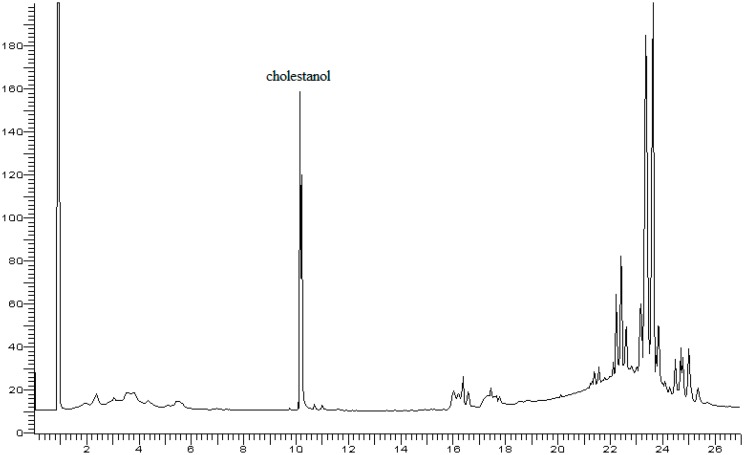
Gas chromatogram of fat extracted from egg yolk with addition of cholestanol as an internal standard (IS). Carrier flow: 2.0 mL/min.

**Figure 5:**
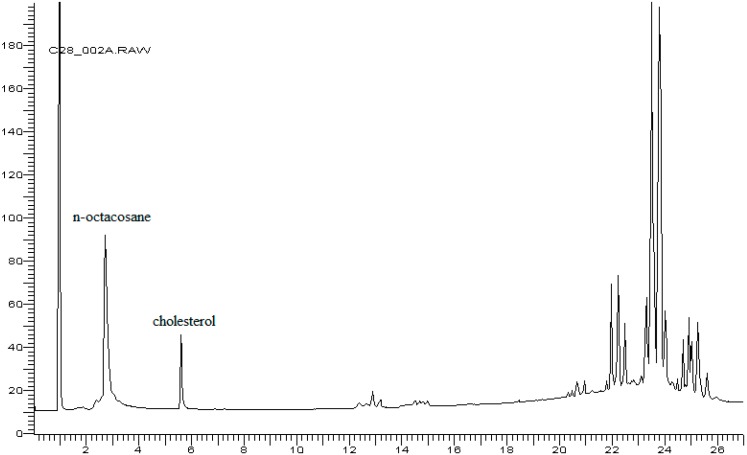
Gas chromatogram of fat extracted from egg yolk with the addition of n-octacosane. Carrier flow: 2.0 mL/min.

The use of the internal standard squalene in place of cholestanol was indispensable when using the polar 65% diphenyl-35% dimethylsilicone column rather than a non-polar column such as 5% diphenyl 95% dimethylsilicone because the latter compound was not sufficiently separated from the cholesterol peak (Figure 4). However, the attempt to use n-octacosane did not lead to a positive result, as its non-polar nature meant that it did not elute properly on the polar column (Figure 5). Instead, squalene, a very stable compound in the absence of oxygen, offered the advantages of being a compound that is stable at room temperature and that elutes on a polar column at approximately the same elution temperature as cholesterol does; it was well separated from cholesterol, and it possesses a comparable number of carbon atoms to cholesterol. All of these features make squalene the optimal internal standard for cholesterol analysis when using a polar stationary phase. The addition of the internal standard allowed accurate measurement of cholesterol; in this way, the measurement was independent of the natural variability of the cholesterol and β-sitosterol content in eggs (Muntoni’s method). The natural variability of cholesterol content in eggs was reported in a previous paper and was in the range of 120–193 mg/egg (average value: 157 ± 3 mg/egg) [11]. This variability was not large, and the use of internal standards guaranteed a good value for the cholesterol added to pasta. In Muntoni’s method, gravimetric determination of cholesterol was affected by larger error.

The analyses of samples prepared in the laboratory gave a recovery for cholesterol of 98.7% ± 0.8%. This result was averaged on the analyses of four samples analysed in triplicate. This result guarantees good recovery for the cholesterol and very low standard deviation.

The fat extracted from the four commercial samples of egg pasta following the official extraction procedure was subjected to gas chromatographic analysis for the determination of cholesterol content as reported in the experimental section. The cholesterol content values are given in Table 1. From the cholesterol values obtained per kilogram of semolina, it was possible to trace a range of added egg values, taking as a reference the cholesterol content in the egg range of 175–250 mg per egg. These values provide the minimum and the maximum value of eggs found experimentally in the literature.

As seen from Table 1, all four commercial egg paste samples exceed the lower limit imposed by current legislation, which includes the addition of at least four eggs per kilogram of semolina. It should be emphasized that sample 3 was labelled with an added egg value of approximately 8, while the value found experimentally was in the range 4.4–6.3. However, this discrepancy was only one case. There was no association with high cholesterol content and standard deviation. The proposed method is applicable in the range 0–2000 ppm, covering the addition of 12 eggs per kg of semolina. Given the great variability of the egg compositional parameters, not all components will be within the limits imposed by law, even in the absence of fraud. However, it is desirable that the parameters provided by law and the industrial procedures for the preparation of the egg paste take into account the new findings and current analytical possibilities that ensure total control of the process by performing upstream analysis on both the product and the semolina to give better control of the number of eggs added to the pasta. On top of these considerations must be added the practical difficulty of obtaining homogeneous mixing of the materials in very short processing times; the non-homogeneity of the product adds yet another variable that leads non-homogeneous finished product lots in the fundamental components. These results led to the conclusion that the determination of the number of eggs in egg pasta using the internal standard method was more accurate than the determination based on the ratio between cholesterol and β-sitosterol or the ratio between cholesterol and campesterol. The former approach provides operators within this sector an accurate method to determine the number of eggs contained in special pasta, both in the preparation phase and the analysis phase. The law regulating egg pasta indicates that the content of total sterols in pasta made with a minimum of four eggs must not be below 0.145 g per parts per hundred dry matter. If the average egg value that has been reported several times in the recent literature of 157 ± 3 mg/egg [11] is accepted and an intake of sterols of 100 mg/kg of semolina is considered, the total content of sterols in the dry pasta should be 0.937 g/kg or 0.0937 g as a percentage of dry matter. These results led to the conclusion that the legal limits should be revised in light of the results obtained recently with respect to the cholesterol content of eggs. The accuracy of the method was evaluated with a recovery test and assessments of repeatability and linearity. The recovery test was performed by spiking the blank pasta matrix with the appropriate amount of cholesterol standard (1000 mg/kg) before the sample extraction step. The recovery of cholesterol was 98.7% ± 0.8%, which could be considered total recovery. The concentrations 0.05 and 2.5 g/kg correspond to the minimum and maximum average concentrations of the egg pasta products, respectively. The spikes at each concentration were prepared in triplicate. The linearity of the method was characterized by the correlation coefficient (*R*^2^ < 0.995), and the linearity range was between 5.0 and 6000 mg/L (*n* = 3). Finally, the two lyophilized egg yolk samples analysed for cholesterol content showed a quantity of cholesterol of 1980 ± 50 mg/kg and 1850 ± 50 mg/kg, respectively. The cholesterol content found in the lyophilized egg was in agreement with previous results reported in the literature. This result could be explained by the fact that the lyophilizate was a homogeneous substance, and this guaranteed an equal distribution of cholesterol in all parts. Therefore, using the lyophilized egg in the preparation of the pasta, the distribution of cholesterol in the pasta was more homogeneous, and for this reason, the determination of the added eggs was more accurate.

In summary, by applying this analytical determination to fat derived from special pasta, this method allowed us to trace the number of eggs added per kg of semolina. The same analytical procedure enabled the determination of the cholesterol content in the lyophilized yolk; the obtained value, using an accurate average content of cholesterol in eggs, could be traced back to the number of eggs from which a certain amount of lyophilized product was derived, allowing the addition of the appropriate quantity of lyophilized egg as an alternative to fresh eggs. Finally, due to the large amount of cholesterol extracted from eggs and egg pasta, it was not necessary to determine the LOD and LOQ. In any case, these parameters were of the order of 10 ppm, a very low quantity that guarantees accuracy for the determination of the cholesterol content at the level of 500–1000 ppm. The recovery for the proposed method was 98.7%. Accordingly, researchers who apply this procedure can achieve simple, rapid analysis.

## 4. Conclusions

The new method proposed in this paper enabled the determination of the cholesterol content in egg pasta quickly and accurately using a very simple analytical procedure based on GC-FID analysis. From the content of cholesterol, it was possible to determine the number of eggs added to the pastas using a mean cholesterol content in eggs of 157 ± 3 mg/egg. The same procedure was also used, with the exception that the Muntoni method, which is based on the ratio between cholesterol and β-sitosterol and/or between cholesterol and campesterol, was applied. The analysis of the triglyceride composition of the fat was made possible by using a more polar GC capillary column (RTX 65 TG-HT), which led to good separation on the polar stationary phase. This analysis could allow the determination of fraud based on the addition of cholesterol as an alternative to the eggs and, at the same time, allow the evaluation of the quality of fat added, thus avoiding possible fraud resulting from the addition of foreign fat to the egg, a determination that would not have been possible using weight analysis or non-polar capillary columns.

## Figures and Tables

**Table 1:** Comparison of number of eggs declared on the label and number of eggs experimentally by the determination of cholesterol.

Commercial Samples	Percentage of Added Eggs (Label)	Egg Number/kg (*) (Declared)	Cholesterol (mg/kg)	No. Determined Eggs (Min–Max)
Sample 1	19.36%	4.8	1025	4.1–5.9
Sample 2	20.30%	5.1	1050	4.2–6.0
Sample 3	28.30%	7.9	1100	4.4–6.3
Sample 4	23.87%	6.3	1200	4.8–6.9

(*) To obtain number of eggs added per kilogram of semolina, the quantity by weight of eggs added to one kilogram of pasta extracted from the percentage declared on the label shall be reduced to 1000 g; the last obtained value is divided by its one-thousandth complement (representing the quantity to which the calculated quantity of eggs has been added), and the result is multiplied by one thousand to obtain the quantity of eggs added to a kilogram of semolina. This quantity obtained is finally divided by the legislative reference weight of an egg equal to 50 g, thus obtaining the number of eggs added to one kilogram of semolina.

## References

[1] Kill R., Turnbull K. (2008). Pasta and Semolina Technology.

[2] Sissons M. (2008). Role of durum wheat composition on the quality of pasta and bread. Food.

[3] Fuad T., Prabhasankar P. (2010). Role of ingredients in pasta product quality: A review on recent developments. Crit. Rev. Food Sci..

[4] Giacco R., Vitale M., Riccardi G., Caballero B., Finglas P., Toldrá F. (2016). Pasta: Role in diet. The Encyclopedia of Food and Health.

[5] Presidential Decree (2001). Decree of the President of the Republic (DPR) of Italy.

[6] Cunniff P., George W.L. (2016). Official Methods of Analysis of AOAC International.

[7] AACC International (1999). Methods 44-15.02, 66–50.01 and 76-31.01. Approved Methods of Analysis.

[8] Muntoni F., Tiscornia E., Tassi-Micco C. (1966). La gas cromatografia nella chimica dei cereali. Nota IV: II dosaggio delle uova nelle paste alimentari. Rassegna Diritto Tecnica Della Alimentazione.

[9] Dinh T., Thompson L. (2016). Cholesterol: Properties, processing effects, and determination. Encycl. Food Health.

[10] Herron K.L., Fernandez M.L. (2004). Are the current dietary guidelines regarding egg consumption appropriate?. J. Nutr..

[11] Naviglio D., Gallo M., Le Grottaglie L., Scala C., Ferrara L., Santini A. (2012). Determination of cholesterol in Italian chicken eggs. Food Chem..

[12] USDA (United Stated Department of Agriculture) (2000). Nutrient Database for Standard Reference.

[13] Mozaffarian D. (2016). Dietary and policy priorities for cardiovascular disease, diabetes, and obesity: A comprehensive review. Circulation.

[14] Djoussé L., Kamineni A., Nelson T.L., Carnethon M., Mozaffarian D., Siscovick D., Mukamal K.J. (2010). Egg consumption and risk of type 2 diabetes in older adults. Am. J. Clin. Nutr..

[15] Fernandez M.L. (2010). Effects of eggs on plasma lipoproteins in healthy populations. Food Funct..

[16] Ballesteros M.N., Valenzuela F., Robles A.E., Artalejo E., Aguilar D., Andersen C.J., Valdez H., Fernandez M.L. (2015). One egg per day improves inflammation when compared to an oatmeal-based breakfast without increasing other cardiometabolic risk factors in diabetic patients. Nutrients.

[17] Missimer A., DiMarco D., Vergara-Jimenez M., Murillo G., Creighton B., Andersen C., Fernandez M.L. (2015). Intake of 2 eggs or oatmeal for breakfast does not increase biomarkers for heart disease while eggs improve liver enzymes and raise HDL cholesterol in young healthy individuals. FASEB J..

[18] DiMarco D.M., Missimer A., Murillo A.G., Lemos B.S., Malysheva O.V., Caudill M.A., Blesso C.N., Fernandez M.L. (2017). Intake of up to 3 eggs/day increases HDL cholesterol and plasma choline while plasma trimethylamine-N-oxide is unchanged in a healthy population. Lipids.

[19] Blesso C.N., Andersen C.J., Bolling B.W., Fernandez M.L. (2013). Egg intake improves carotenoid status by increasing plasma HDL cholesterol in adults with metabolic syndrome. Food Func..

[20] Naviglio D., Raia C. (2003). Application of a HRGC method on capillary column Rtx 65-TG for triglyceride analysis to monitor butter purity. Anal. Lett..

[21] Christie W.W. (1993). Preparation of ester derivatives of fatty acids for chromatographic analysis. Adv. Lipid Method.

[22] Nota G., Naviglio D., Romano R., Luongo D., Spagna Musso S. (1994). Un metodo rapido per la determinazione del numero delle uova nelle paste speciali. Riv. Ital. Sostanze Grasse.

[23] Boselli E., Velazco V., Caboni M.F., Lercker G. (2001). Pressurized liquid extraction of lipids for the determination of oxysterols in egg-containing food. J. Chromatogr..

[24] Dinh T.T., Thompson L.D., Galyean M.L., Brooks J.C., Patterson K.Y., Boylan L.M. (2011). Cholesterol content and methods for cholesterol determination in meat and poultry. Compr. Rev. Food Sci. Food Saf..

[25] Carrapiso A.I., García C. (2000). Development in lipid analysis: Some new extraction techniques and in situ transesterification. Lipids.

[26] Čížková H., Prokorátová V., Voldřich M., Kvasnička F., Soukupová V. (2004). Determination of egg content in pasta. Czech J. Food Sci..

[27] Fodor M., Woller A., Turza S., Szigedi T. (2011). Development of a rapid, non-destructive method for egg content determination in dry pasta using FT-NIR technique. J. Food Eng..

[28] Bevilacqua M., Bucci R., Materazzi S., Marini F. (2013). Application of near infrared (NIR) spectroscopy coupled to chemometrics for dried egg-pasta characterization and egg content quantification. Food Chem..

[29] Albuquerque T.G., Oliveira M.B.P., Sanches-Silva A., Costa H.S. (2016). Cholesterol determination in foods: Comparison between high performance and ultra-high performance liquid chromatography. Food Chem..

[30] Langella C., Naviglio D., Marino M., Gallo M. (2015). Study of the effects of a diet supplemented with active components on lipid and glycemic profiles. Nutrition.

[31] Madzlan K. (2008). Determination of cholesterol in several types of eggs by gas chromatography. J. Trop. Agric. Food Sci..

[32] Dinh T.T., Thompson L.D., Galyean M.L., Brooks J.C.L., Boylan M.L. (2012). Determination of total cholesterol in meat and poultry by gas chromatography: Single-laboratory validation. J. AOAC Int..

[33] Egressy-Molnár O., Jókai Z. (2011). Development of a GC-based method for the determination of egg content in dried pasta with the focus on method validation. Acta Aliment..

[34] Al-Balaa D., Rajchl A., Grégrová A., Ševčík R., Čížková H. (2014). DART mass spectrometry for rapid screening and quantitative determination of cholesterol in egg pasta. J. Mass Spectrom..

[35] Naviglio D., Dellagreca M., Ruffo F., Andolfi A., Gallo M. (2017). Rapid analysis procedures for triglycerides and fatty acids as pentyl and phenethyl esters for the detection of butter adulteration using chromatographic techniques. J. Food Qual..

